# Patient Satisfaction and Trust in Telemedicine During the COVID-19 Pandemic: Retrospective Observational Study

**DOI:** 10.2196/28589

**Published:** 2021-04-22

**Authors:** Sharon Orrange, Arpna Patel, Wendy Jean Mack, Julia Cassetta

**Affiliations:** 1 Keck School of Medicine of USC University of Southern California Los Angeles, CA United States

**Keywords:** telemedicine, patient satisfaction, COVID-19, health services research, health policy, health care delivery, physicians, medicine

## Abstract

**Background:**

Los Angeles County is a hub for COVID-19 cases in the United States. Academic health centers rapidly deployed and leveraged telemedicine to permit uninterrupted care of patients. Telemedicine enjoys high patient satisfaction, yet little is known about the level of satisfaction during a crisis and to what extent patient- or visit-related factors and trust play when in-person visits are eliminated.

**Objective:**

The aim of this study is to examine correlates of patients’ satisfaction with a telemedicine visit.

**Methods:**

In this retrospective observational study conducted in our single-institution, urban, academic medical center in Los Angeles, internal medicine patients aged ≥18 years who completed a telemedicine visit between March 10th and April 17th, 2020, were invited for a survey (n=1624). Measures included patient demographics, degree of interpersonal trust in patient-physician relationships (using the Trust in Physician Scale), and visit-related concerns. Statistical analysis used descriptive statistics, Spearman rank-order correlation, and linear and ordinal logistic regression.

**Results:**

Of 1624 telemedicine visits conducted during this period, 368 (22.7%) patients participated in the survey. Across the study, respondents were very satisfied (173/365, 47.4%) or satisfied (n=129, 35.3%) with their telemedicine visit. Higher physician trust was associated with higher patient satisfaction (Spearman correlation *r*=0.51, *P*<.001). Visit-related factors with statistically significant correlation with Trust in Physician score were technical issues with the telemedicine visit (*r*=–0.16), concerns about privacy (*r*=–0.19), concerns about cost (*r*=–0.23), satisfaction with telemedicine convenience (*r*=0.41), and amount of time spent (*r*=0.47; all *P*<.01). Visit-related factors associated with patients’ satisfaction included fewer technical issues (*P*<.001), less concern about privacy (*P*<.001) or cost (*P*=.02), and successful face-to-face video (*P*<.001). The only patient variable with a significant positive association was income and level of trust in physician (*r*=0.18, *P*<.001). Younger age was associated with higher satisfaction with the telemedicine visit (*P*=.005).

**Conclusions:**

There have been calls for redesigning primary care after the COVID-19 pandemic and for the widespread adoption of telemedicine. Patients’ satisfaction with telemedicine during the COVID-19 pandemic is high. Their satisfaction is shaped by the degree of trust in physician and visit-related factors more so than patient factors. This has widespread implications for outpatient practices and further research into visit-related factors and the patient-provider connection over telemedicine is needed.

## Introduction

On March 11, 2020, the World Health Organization declared the COVID-19 outbreak a pandemic; thereafter, telemedicine—particularly video consultation—was promoted and scaled up to reduce the risk of transmission [1,2]. A few months later, Los Angeles became the county with the highest number of COVID-19 cases in the United States [3,4]. To prioritize public health, our academic health center rapidly deployed and leveraged telemedicine in response to the COVID-19 pandemic, permitting uninterrupted care of our patients [5]. We transitioned all clinic encounters as of March 16, 2020, to telemedicine, defined here as synchronous video or telephone visits [6,7].

Studies have shown that telemedicine visits enjoy high patient satisfaction [8,9]. Still, little is known about patient satisfaction with their primary care provider during a pandemic when patients have little choice but to seek remote care. Historically, correlates of patient satisfaction with telemedicine represent patients who have chosen that platform and thus are skewed toward a younger, female, and underinsured or uninsured population [10,11]. Additionally, patient satisfaction with direct-to-consumer telemedicine has been assessed with little or no previous doctor-patient relationship or coordination with the patients’ primary care provider [12]. Patient trust in their provider, an essential foundation for fostering patient satisfaction, has not been well studied in this type of remote care setting [13].

Rapid implementation of telemedicine within practices has been proposed to properly care for patients during the pandemic and beyond [14,15]. With the tremendous advances in telemedicine since COVID-19, determining factors correlated with satisfaction carries widespread implications for outpatient medicine and efforts to establish a framework for satisfying telemedicine visits. These findings are crucial for providers in adopting telemedicine as an element of the patient care continuum.

We captured 6 weeks of telemedicine visits in our primary care practice to explore the relationship between trust and patient satisfaction during a telemedicine visit, which has received little attention [16-18]. We examined whether patient factors, visit-associated factors, and the degree of “trust in provider” contributed to a satisfying telemedicine visit. We hypothesized that patient satisfaction with a telemedicine visit would be positively related to the degree of trust in the provider, patient-specific factors, and ease of use of the telemedicine platform.

## Methods

Keck Medical Center is a large academic medical center located in Los Angeles. Inpatient services are provided at our institution at Keck Medical Center and USC Verdugo Hills Hospital, while outpatient services are provided at Keck Medical Center Outpatient facilities; both institutions share the same providers.

### Data Source

Upon providing informed consent, the respondent was invited to complete a questionnaire provided by electronic survey. To explore the degree to which “trust in physician” correlates with satisfaction with telemedicine, we used a previously validated measure, the 11-question Trust in Physician Scale [19], to assess interpersonal trust in patient-physician relationships. Responses were scored on a 5-point Likert scale and higher scores indicated higher levels of trust (scale range 11-55).

Telemedicine visit–related issues and concerns including cost, privacy, convenience, technical issues, and time were assessed using a 5-item Likert scale. Responses ranged from 1-5 and higher scores indicated higher levels of agreement/satisfaction.

Satisfaction with the telemedicine visit was measured using the statements “I look forward to using telehealth in the future” (yes/no) and “To what extent were you satisfied with your visit?” (5-item Likert scale).

Respondents were also asked several questions about their demographics and health status.

### Study Population

We performed a retrospective study of patients aged 18 years and older who had one or more telemedicine visits with a provider in the internal medicine department at the Keck Medical Center between March 10th and April 17th, 2020. This timing corresponds with a Keck Medical Center mandate to shift the majority of outpatient care from in-person to telemedicine visits. A total of 1744 patients had an encounter with our internal medicine providers during that time, and a link to a survey was successfully emailed to 1624 patients (93%). Data were collected in the fall of 2020. To be eligible to participate, the respondent had to have a telemedicine visit with one of our primary care providers. With a final sample size of 368 responders (22.7%), the attained sample size provided 80% statistical power to detect correlations of 0.14 and higher. All patients during the study period were invited to a video-enabled telehealth visit; of the 368 responders, 284 (77.4%) used video with their telehealth visit and the rest were telephone consultations. The study database in REDCap used the survey feature; all surveys were completed anonymously, and no personal health information or personally identifiable information on survey respondents was collected, in compliance with the Health Insurance Portability and Accountability Act (HIPAA). Nonresponders were similar in gender to responders (60.3% female versus 64.4% female), but responders were older than nonresponders by an average of 4.5 years (*P*<.001).

### Statistical Analysis

Descriptive statistics were used to summarize visit-related concerns, patient characteristics, and satisfaction with the telemedicine visit. Variables were summarized as frequency and percentages for categorical variables and median and IQR for continuous variables.

The association of the Likert scale satisfaction item with trust in physician was evaluated with a Spearman rank-order correlation. The median (IQR) Trust in Physician Scale score is presented by level of patient satisfaction.

Associations of patient- and visit-related factors with Trust in Physician score and patient satisfaction used Spearman rank-order correlation, linear regression, and ordinal logistic regression (ordinal patient satisfaction dependent variable). Patient- and visit-related factors found in a linear regression analysis to be associated with the Trust in Physician score were included as independent variables to obtain an estimate and test of the adjusted association of trust with satisfaction with the telemedicine encounter.

## Results

### Preliminary Analysis

A link to a survey was emailed to 1624 patients; there were 368 respondents. The characteristics of the sample (N=368) are described in Table 1. The sample was primarily female and White, with a mean age of 55.8 (SD 16.0) years. Respondents evaluated their current health as fair to good.

Across the study, respondents were very satisfied (173/365, 47.4%) or satisfied (n=129, 35.3%) with their telemedicine visit, and 77.3% (279/361) reported that they “look forward to using telehealth in the future.” Table 2 describes the visit characteristics of the sample. Respondents tended not to worry about privacy or the cost of the telemedicine visit. The majority of patients (284/367, 77.4%) used video with their telehealth visit, while the rest were telephone consultations. Face-to-face video rather than telephone alone was preferred by most respondents, with 67.7% (243/359) strongly agreeing/agreeing it was important. Almost one-third of patients (114/365, 31.3%) had technical issues during the visit, yet 63 were resolved during the telemedicine visit. Notably, despite technical challenges, the convenience of telehealth was supported by 55.7% (204/366) and 32.8% (n=120) of patients who strongly agreed and agreed the telehealth visit was convenient, respectively. There was high satisfaction among our respondents with the amount of time spent and 90.1% (327/363) strongly agreed or agreed that the amount of time spent with the provider was adequate. Patients did not appear to have privacy concerns, with 28.8% (105/365) strongly disagreeing and 40% (n=146) disagreeing that they were “concerned about privacy.”

A summary of results from respondents to the 11-point Trust in Physician Scale appears in Table 3. Respondents overwhelmingly agreed with the statement “I trust my doctor’s judgments about my medical care” and that their doctor “is a real expert in taking care of medical problems.”

**Table 1:** Patient characteristics.

Characteristics		Values
Age in years (n=365), median (IQR)		57 (43-68)
Hispanic (n=366), n (%)		96 (26.2)
**Race (n=348), n (%)**		
	White	262 (70)
	Black or African American	25 (7.2)
	American Indian or Alaskan Native	7 (2)
	East Asian	28 (8.1)
	Southeast Asian	14 (4)
	Asian Indian	3 (0.9)
	Native Hawaiian or Pacific Islander	3 (0.9)
	Some other race	32 (9.2)
Female (n=364), n (%)		239 (66)
**Education (n=364), n (%)**		
	Less than high school	10 (2.8)
	High school degree or equivalent	14 (3.9)
	Some college but not degree	67 (18.4)
	Bachelor’s degree	109 (30)
	Graduate degree	164 (45.1)
**Current health (n=365), n (%)**		
	Excellent	46 (12.6)
	Good	196 (53.7)
	Fair	98 (26.9)
	Poor	25 (6.9)
**Income in US $ (n=364), n (%)**		
	0-19,999	29 (8)
	20,000-39,999	17 (4.7)
	40,000-59,999	22 (6)
	60,000-79,999	37 (10.1)
	80,000-99,999	24 (6.6)
	100,000-119,999	21 (5.6)
	120,000-139,999	21 (5.6)
	140,000-159,999	21 (5.6)
	160,000-179,999	11 (3)
	180,000-199,999	13 (3.6)
	200,000 or more	78 (21.4)
	Prefer not to answer	70 (19.2)

**Table 2:** Visit characteristics.

Characteristics		Participants, n (%)	Median (IQR)	
Used video with your telehealth visit (n=367)		284 (77.4)	N/A^a^	
**Did you experience significant technical issues before or during your visit? (n=365)**				N/A
	Yes	51 (14)		
	Yes, but it was resolved during telehealth visit	63 (17.3)		
	No	251 (69)		
**What sort of technical issues did you have? (n=110)**				N/A
	Sound was not working	13 (11.8)		
	Video was not working	38 (34.5)		
	I was able to connect, but via different telehealth sources	32 (39.1)		
	Other issues	27 (24.6)		
**The telehealth visit was convenient (n=366)**				5 (4-5)
	Strongly disagree	7 (1.9)		
	Disagree	11 (3)		
	Neither agree nor disagree	24 (6.6)		
	Agree	120 (32.8)		
	Strongly agree	204 (55.7)		
**The amount of time spent was adequate (n=363)**				5 (4-5)
	Strongly disagree	5 (1.4)		
	Disagree	9 (2.5)		
	Neither agree nor disagree	22 (6.1)		
	Agree	134 (36.9)		
	Strongly agree	193 (53.2)		
**I was concerned about privacy (n=365)**				2 (1-3)
	Strongly disagree	105 (28.8)		
	Disagree	146 (40)		
	Neither agree nor disagree	63 (17.3)		
	Agree	28 (7.7)		
	Strongly agree	23 (6.3)		
**Having face-to-face video was important (n=359)**				4 (3-5)
	Strongly disagree	7 (2)		
	Disagree	22 (6.1)		
	Neither agree nor disagree	87 (24.2)		
	Agree	108 (30.1)		
	Strongly agree	135 (37.6)		
**I was worried how much my telehealth visit would cost (n=363)**				2 (2-3)
	Strongly disagree	83 (22.9)		
	Disagree	114 (31.4)		
	Neither agree nor disagree	112 (30.9)		
	Agree	36 (9.9)		
	Strongly agree	18 (4.5)		
I look forward to using telehealth in the future (n=361)		279 (77.3)	N/A	
**To what extent were you satisfied with your visit (n=365)**				N/A
	Very unsatisfied	10 (2.7)		
	Unsatisfied	14 (3.8)		
	Neutral	39 (10.7)		
	Satisfied	129 (35.3)		
	Very satisfied	173 (47.4)		
**Did you recover from your illness? (n=312)**				N/A
	Yes	12 (3.9)		
	Yes, but I required more than one telehealth visit	12 (3.9)		
	No, I was seen in an urgent care clinic/emergency room	70 (22.4)		
	No, I was sent to the Keck Medical evaluation tent or Evaluation and Treatment Center	218 (69.9)		

^a^N/A: not applicable.

**Table 3:** Trust in Physician Scale responses.

Statements		Participants, n (%)	Median (IQR)	
**I doubt my doctor really cares about me as a person (n=366)**				1 (1-2)
	Strongly disagree	202 (55.2)		
	Disagree	104 (28.4)		
	Neither agree nor disagree	40 (10.9)		
	Agree	8 (2.2)		
	Strongly agree	12 (3.3)		
**My doctor is usually considerate of my needs and puts them first (n=365)**				5 (4-5)
	Strongly disagree	7 (1.9)		
	Disagree	4 (1.1)		
	Neither agree nor disagree	32 (8.8)		
	Agree	131 (35.9)		
	Strongly agree	191 (52.3)		
**I trust my doctor so much I always try to follow his/her advice (n=365)**				4 (4-5)
	Strongly disagree	6 (1.6)		
	Disagree	2 (0.5)		
	Neither agree nor disagree	33 (9)		
	Agree	152 (41.6)		
	Strongly agree	172 (47.1)		
**If my doctor tells me something is so, then it must be true (n=363)**				4 (3-4)
	Strongly disagree	8 (2.2)		
	Disagree	23 (6.3)		
	Neither agree nor disagree	117 (32.2)		
	Agree	153 (42.2)		
	Strongly agree	62 (17.1)		
**I sometime distrust my doctor’s opinion and would like a second one (n=362)**				2 (2-3)
	Strongly disagree	82 (22.7)		
	Disagree	152 (42)		
	Neither agree nor disagree	85 (23.5)		
	Agree	35 (9.7)		
	Strongly agree	8 (2.2)		
**I trust my doctor’s judgements about my medical care (n=362)**				4 (4-5)
	Strongly disagree	5 (1.4)		
	Disagree	3 (0.8)		
	Neither agree nor disagree	25 (6.9)		
	Agree	167 (46.1)		
	Strongly agree	162 (44.8)		
**I feel my doctor does not do everything he/she should for my medical care (n=363)**				2 (1-2)
	Strongly disagree	148 (40.7)		
	Disagree	137 (37.7)		
	Neither agree nor disagree	44 (12.1)		
	Agree	24 (6.6)		
	Strongly agree	10 (2.8)		
**I trust my doctor to put my medical needs above all other considerations when treating my medical conditions (n=362)**				4 (4-5)
	Strongly disagree	4 (1.1)		
	Disagree	8 (2.2)		
	Neither agree nor disagree	47 (13)		
	Agree	151 (41.7)		
	Strongly agree	152 (42)		
**My doctor is a real expert in taking care of medical problems (n=363)**				4 (4-5)
	Strongly disagree	3 (0.8)		
	Disagree	4 (1.1)		
	Neither agree nor disagree	51 (14)		
	Agree	154 (42.2)		
	Strongly agree	151 (41.6)		
**I trust my doctor to let me know if a mistake was made about my treatment (n=362)**				4 (4-5)
	Strongly disagree	4 (1.1)		
	Disagree	8 (2.2)		
	Neither agree nor disagree	54 (14.9)		
	Agree	158 (43.7)		
	Strongly agree	138 (38.1)		
**I sometimes worry that my doctor may not keep the information we discuss totally private (n=365)**				1 (1-2)
	Strongly disagree	199 (54.5)		
	Disagree	115 (31.5)		
	Neither agree nor disagree	47 (12.9)		
	Agree	3 (0.8)		
	Strongly agree	1 (0.3)		
Physician trust total score^a^ (n=345)				46 (42-51)

^a^Physician trust total score generated by the sum of 11 items from the physician trust survey. Highest possible score=55; mean 45 (SD 6.5).

### Trust in Physician and Satisfaction With Telemedicine Visit

Higher physician trust was associated with higher patient satisfaction with the telemedicine visit. Results of the Spearman correlation indicated that there was a significant positive association between the degree of patients’ trust in physician and satisfaction with their telemedicine visit (*r*=0.51, *P*<.001).

### Patient Factors and Trust in Physician

Overall, patient factors including age (*r*=–0.01, *P*=.81), level of education (*r*<0.01, *P*=.99), and current health status (*r*=–0.01, *P*=.78) were not significantly correlated with level of trust in their physician. There was, however, a significant positive association between income and level of trust in physician (*r*=0.18, *P*<.001).

### Visit-Related Factors and Trust in Physician

In contrast to patient factors, several visit-related factors showed a significant correlation with Trust in Physician score. Respondents who did not have technical issues (*r*=–0.16, *P*=.002), concerns about privacy (*r*=–0.19, *P*<.001), or concerns about the cost (*r*=–0.23, *P*<.001) had a higher degree of trust in their physician. Those who agreed face-to-face video was important (*r*=0.23, *P*<.001), liked the convenience (*r*=0.41, *P*<.001), and were satisfied with the amount of time spent (*r*=0.47, *P*<.001) also showed a higher degree of trust in their physician.

### Patient Factors and Satisfaction With Telemedicine Visit

Patient factors including gender (*P*=.67), education (*P*=.82), income (*P*=.14), and current health (*P*=.18) were not associated with satisfaction with their telemedicine visit. Age was the only significant factor associated with satisfaction, with a younger median age of 54 (IQR 42-64) years among those who were very satisfied compared to a median age of 60 (IQR 50-69) years among those who were unsatisfied or neutral (likelihood ratio *P*=.005 with ordinal logistic regression).

### Visit-Related Factors and Satisfaction With Telemedicine Visit

Evaluated by ordinal logistic regression, all visit-related factors were associated with patient satisfaction with their telemedicine visit. Fewer technical issues (*P*<.001), acknowledging the convenience (*P*<.001), appreciating the amount of time spent (*P*<.001), fewer concerns about privacy (*P*<.001) and cost (*P*=.02), and successful face-to-face video (*P*<.001) were all significantly associated with a satisfying telemedicine visit.

## Discussion

The COVID-19 pandemic poses unique challenges to health care delivery, especially for those in primary care. Patient fear surrounding COVID-19 has disrupted patients’ normative expectations toward their doctors (and vice versa), creating more complex trust relationships. Prior studies have shown patients prefer telemedicine with a doctor with whom they have an established relationship [20]. When it comes to specialist referral, trust and confidence in one’s primary care provider are crucial to creating a satisfying experience [21,22].

Telemedicine, particularly video consultation, has been rapidly implemented to reduce the risk of transmission. Before this historic period, studying telemedicine satisfaction would have posed a self-selection bias, which the pandemic mostly eliminated due to institutional and patient health precautions early on. Correlates of patient satisfaction aid to inform and further educate practices adopting telemedicine and the pandemic provides a unique opportunity to evaluate those visits and factors affecting satisfaction.

Patients’ trust in their physician, telemedicine services, and willingness to rely on such a health service for care during a pandemic has not previously been described. Researchers have given little attention to which factors contribute to trust in a telemedicine visit, a unique situation made more difficult during the pandemic. A previously reported study on the use of telehealth visits for anticoagulation management found trust in the technology, trust in health care professionals, and trust in the treatment affected trust in the telemedicine service [23]. The rapid transition to telemedicine requires providers and patients to transition to a new normal that includes communicating via telephone or video. For providers, this means developing skills in building trust, counseling, empathy, “modified” physical exams, and diagnosis using the telemedicine platform. Prior telemedicine studies include a level of self-selection, yet provide some insight into the importance of trust in provider for telemedicine visits. In one study, patients who chose a virtual follow-up over an in-person visit spoke of the importance of an existing doctor-patient relationship and having already had previous consultations with that same person before the follow-up video consultation [24].

Recent suggestions on fostering human connection have focused primarily on telemental health, with tips provided for enhancing virtual connections, such as being “present,” identifying needs, listening, responding with empathy, and sharing information [25]. Empirical evidence in this area is sparse and achieving greater clarity about factors contributing to a satisfying telemedicine visit would help health care providers better anticipate patients’ needs.

Our study provides new insights into the reasons for a satisfying telemedicine visit when an established relationship with the provider or practice exists. Consistent with our hypothesis and using our patients’ experience at the onset of the COVID-19 pandemic, we found that trust in physician, as assessed using the 11-question Trust in Physician Scale, was correlated with higher patient satisfaction in telemedicine visits. Patients who trust their doctor and try to follow his/her advice, trust their doctor’s judgment about medical care, and believe their doctor will let them know if a mistake was made about their treatment were more likely to be satisfied with a telemedicine visit and wanted to use the platform again. These findings suggest a significant role in provider engagement, fostering human connection, and strengthening the patient-physician attachment. Higher physician trust was positively correlated with greater patient satisfaction with telemedicine.

Furthermore, factors related to the visit, including privacy, cost, convenience, and time, were associated with higher satisfaction and higher trust in physicians. Our findings suggest that ease of use with fewer technical issues and video-enabled visits result in higher patient satisfaction and higher trust in physician. At our institution, test calls before initial sessions help evaluate the level of technological support a patient needs for the upcoming telemedicine visit.

Our findings support a role for continued improvement in training and operational issues in telemedicine.

While the study group was mostly White, high-income, and well-educated, our study did not find evidence that patient-related factors in this sample play a significant role in trust in physician or the likelihood of a satisfying telemedicine visit. Patient income was positively associated with level of trust; this association has been reported for in-person care, where lower physician trust is seen with lower income [21]. Our study found higher income correlated with a higher level of trust in physician, which was positively associated with patient satisfaction with telemedicine. Consistent with prior research that shows younger patients, perhaps due to higher eHealth literacy, have higher acceptance of the telemedicine platform [26,27], we also found that younger age correlated with a satisfying telemedicine visit. Our predominantly younger White female population is consistent with prior studies on the acceptance of telemedicine [24,27].

This study has several limitations. First, this was a retrospective study with no comparison to in-person visit satisfaction during the same period or before the pandemic. We did not feel the pandemic’s challenging situation, which did not allow for the option of in-person visits, could be compared to prior visits. As the pandemic lifts, this would be something evaluated in future studies. Previous studies on the acceptability of video consulting show that even among those who would choose that format again, face-to-face consulting was seen as the gold standard and preferred for both provider and patient for emotionally charged or more challenging consultations [24]. Second, the use of a web-based survey prevents us from recruiting patients without an email address (n=113, 7%), potentially leading to bias toward respondents with higher digital literacy. Third, the response rate to the survey was lower than anticipated (368/1624, 22.7%). We suspect replying to an email survey in the early days of the pandemic presented additional challenges to our patient population who had not necessarily chosen the telemedicine platform. Fourth, respondents were significantly older than our nonresponders (55.8 years versus 51.3 years, *P*<.001), yet while our findings support younger age as a factor correlated with satisfaction with their visit, age was not correlated with trust in physician. Fifth, the Likert-based satisfaction item, although face valid, was not derived from a validated questionnaire. Lastly, as our study population was less ethnically and racially diverse than the overall United States and Los Angeles County population, we could not capture the experiences of underrepresented minorities and underserved communities.

In conclusion, this study suggests most patients are satisfied with telemedicine visits during the COVID-19 pandemic and that trust in physician correlates favorably with patient satisfaction. Trust and satisfaction are shaped by many visit-related factors, including convenience, time spent, and video-enabled encounters, rather than specific patient-related factors. Our study reinforces telemedicine as a new form of health care delivery even in times of uncertainty, supporting our hypothesis that patient satisfaction with a telemedicine visit would be positively related to the degree of trust in the provider and ease of use of the telemedicine platform. Further studies examining patient-physician relationships over telemedicine may better elucidate elements contributing to patients’ trust in their physicians. With calls to promote and scale-up telemedicine in primary care, this will help develop a strategy and operational plans for providers to switch to remote patient care.
